# Perception of Older Thai Adults in a Do-Not-Attempt Resuscitation Order during the COVID-19 Era If Infected with COVID-19

**DOI:** 10.1089/pmr.2021.0084

**Published:** 2022-06-21

**Authors:** Jiraporn Sri-on, Pannawat Wongthanasit, Thitiwan Paksopis, Shan W. Liu, Khemika Rojtangkom, Rasida Ruangsiri

**Affiliations:** ^1^ Geriatric Emergency Medicine Unit, The Department of Emergency Medicine, Vajira Hospital, Navamindradhiraj University, Bangkok, Thailand.; ^2^ The Department of Emergency Medicine, Vajira Hospital, Navamindradhiraj University, Bangkok, Thailand.; ^3^ The Department of Emergency Medicine, Massachusetts General Hospital, Harvard University, Boston, Massachusetts, USA.; ^4^ The Clinical Research Center, Vajira Hospital, Navamindradhiraj University, Bangkok, Thailand.; ^5^ Thai Health Promotion Organization (ThaiHealth), Bangkok, Thailand.

**Keywords:** COVID-19, DNR, end of life, older adults, Thai

## Abstract

**Background:**

During the coronavirus disease 2019 (COVID-19) pandemic, older adults experienced high mortality rates, and their deaths were often preceded by sudden health deterioration and acute respiratory failure. This prompted older adults and their families to make rapid goals-of-care decisions.

**Objective:**

This study aimed at determining the prevalence of and factors associated with COVID-19-related do-not-attempt resuscitation (DNR) decisions among older adults.

**Design:**

This was a cross-sectional population-based survey.

**Setting:**

Well-looking active (mobile) community-dwelling adults aged ≥60 years and residing in the Bangkok district, Thailand, between April and May 2020, were included in this study. We excluded older adults who (1) were unable to speak Thai, (2) had severe cognitive impairment, or (3) were blind or deaf. We interviewed participants about their perceptions regarding end-of-life decisions in case they got infected with COVID-19 and experienced respiratory arrest.

**Results:**

We recruited 848 participants with a mean age of 70.5 (±6.74) years. When asked about their choice, 49.8% chose a DNR status, 44.5% chose full life support, and 5.8% were undecided. The three most common reasons provided by the DNR group for their choice were old age (54.9%), acceptance of death (15.6%), and fear of pain (8.5%).

**Conclusion:**

Almost half of the older Thai adults chose a DNR status for scenarios in which they were infected with COVID-19 and suffered from cardiac arrest during the pandemic period. Future studies should include an in-depth examination of participants' lifestyles, family life expectancy, and religious faith to understand their end-of-life decisions.

## Introduction

As they approach death, older adults often deal with emotional, spiritual, and physical challenges. Most often, there is an increase in medical intervention as death nears.^1^ A U.S. study reported that more than 30% of critically ill older adults are intubated and mechanically ventilated.^2^ Another study reported that 35% of older emergency department (ED) patients died after two to three days of intubation; a rate that was higher among those with cerebrovascular disease, sepsis, and myocardial infarction.^3^

A do-not-attempt resuscitation (DNR) order is a legal order that allows natural death.^4,5^ Steinhauser et al.^5^ found that >90% of patients perceive that at the end of life, individuals need freedom from pain, freedom from shortness of breath, freedom from anxiety, and freedom to be treated as a whole person.

In December 2019, the first case of coronavirus disease 2019 (COVID-19) was detected in China.^6^ Since then, there have been 178 million reported cases and more than 3.8 million deaths globally (reports of June 24, 2021).^7^ In the United States, people aged older than 65 years represented 80% of the deaths.^8^ Older adults are more susceptible to COVID-19 because of the physiological changes and multiple comorbidities associated with aging.^9^ COVID-19 patients who arrive at the ED are often critically ill, and the dying process in COVID-19 patients is different from the natural dying process.

In an event of sudden health deterioration and acute respiratory failure, frail older adults and their families are often required to make rapid decisions under highly stressful circumstances regarding whether or not to pursue all available treatments (sustained life support), regardless of the chance of recovery. Understanding the natural history of COVID-19 and its treatment is important. Further, the limited number of health facilities and personal protection equipment, and the risk of transmitting the infection to health care providers are important factors to consider in the ED. In the early stages of the pandemic, many countries had to deal with a shortage of medical staff and resources.^10,11^

The ED care providers had to make difficult decisions regarding life-sustaining interventions, admissions, and end-of-life decisions.^12^ In New York, toward the end of their lifetime, 17% of older adults infected with COVID-19 or at a high risk of death from COVID-19 did not request life-sustaining treatments, and the prevalence of DNR orders increased to 90% after the patients were visited by the palliative care team.^13–15^

Thailand, a middle-income country, had 3081 cases of COVID-19 infections and 57 deaths in May 2020; however, this increased to more than 2,150,000 cases and 20,997 deaths on December 9, 2021.^16^ These infected cases were mostly concentrated in the central and urban areas of Thailand.^17^ Srinonprasert et al. studied outpatient clinics before the COVID-19 outbreak and found that 59.2% of Thai older adults wished to die at home,^18^ indicating that many of the older Thai adults had already considered DNR orders.

Further, most studies of Thai palliative and end-of-life care have focused on cancers and non-communicable diseases.^19–21^ A knowledge of DNR perceptions would enable families and older adults to make DNR decisions before they get sick. To date, no studies have examined end-of-life code status preferences in the Thai population in the context of COVID-19 infection. This study, therefore, aimed at determining the perceptions of older urban Thai adults regarding DNR orders during the COVID-19 era.

## Methods

### Study design

Between April 1, 2020 and May 31, 2020, we conducted a cross-sectional telephone questionnaire survey in Bangkok, Thailand. This study was part of the prospective cohort study titled “Proposal of modifications to the Bangkok urban health system that would improve the quality of health, independent living, and maintenance of older adults with fall-related trauma (Bangkok Falls Study).”^22^ We enrolled adults aged 60 years and older living in one of the five sub-districts of the Dusit District, Bangkok, Thailand between October 1, 2019, and September 30, 2021.

Summarily, we included adults registered in the Dusit district office who were able to walk and who expected to continue living in that community for at least two years. We excluded older adults who were unable to speak Thai, those with severe cognitive impairment (defined by a scoring >12 points on the six-item cognitive screening test), and those who were blind or deaf. Participants were recruited using the snowball sampling method. This study was approved by our hospital's institutional review board.

The study had three phases of data collection:

Phase 1: Community data collection: The research assistant (RA) team collected data on baseline characteristics, underlying diseases, medications used, Charlson comorbidity indices (CCI),^23^ Barthel activities of daily living (ADL) indices,^24^ mini-nutritional assessment scores,^25^ six-item cognitive impairment test (6-CIT) scores,^26^ World Health Organization quality of life (WHOQOL-BREF-THAI) scores,^27^ caregivers' information, income levels, education levels, and mobility statuses.
Phase 2: Laboratory and clinical examination over a period of one month after the community visit.
Phase 3: The RAs performed telephone follow-ups with participants at 3, 6, and 12 months after their enrollment, and the questionnaire of this project was administered during the third month of the follow-up.

The RAs had bachelor degrees in health science with at least three years' experience in clinical research of older adults, or were nurse practitioners with a minimun of five years' experience in the field of geriatrics.

Because of the outbreak of COVID-19 at that time, the Thailand government announced a lockdown policy for the Thai population. The questionnaires included questions on ADL, awareness of COVID-19 and its routes of transmission, history of traveling during the previous 14 days, reports of COVID-19 cases in the community, end-of-life decisions, and the reasons for requesting or not requesting resuscitation. The time taken to complete the survey was <15 minutes.

Process of survey development

1. Item generation and construction: We followed the questionnaire concept of Catt and Blanchard.^28^ The questionnaire was drafted in the Thai language. We used baseline characteristics, Barthel ADL indices, CCI, and World Health Organization quality-of-life (WHOQOUL-BREF THAI) frailty phenotype^29^ assessments from the community visit data. We added four items about COVID-19; these included awareness of COVID-19, awareness of the routes of transmission, history of traveling within the past 14 days, and awareness of reported cases in the community. The end-of-life questions included (1) the decision participants would make should they contract COVID-19 infection and develop cardiac arrest, and (2) The reason for their decision, irrespective of whether they chose DNR or not (open-ended questions).
2. Pilot testing and clarification: We performed a pilot test of the survey on a group of 10 older adults who visited the ED at that time and adjusted the terms and language in the questionnaire as deemed necessary.

### Measurements

#### Care giver information

It was defined as (1) Had a caregiver or (2) No caregiver. If the participants had a caregiver, we asked the latter about their relationship with the participants: spouses, first-degree relatives, second-degree relatives, etc.

#### Income level

We collected data from the participants regarding their average monthly income in the past year.

#### Travel in the past 14 days

Travel outside the province in the past 14 days.

#### The process of coding the people's narrative responses regarding their choice

Two emergency physicians (EPs) discussed with the first 100 participants and coded their narrative responses regarding end-of-life decisions into one of the following domains: DNR, full life support, and “undecided” decisions. They recorded and coded their responses. After interviewing the first 100 participants, the two EPs separately coded the decisions of the remaining participants. If there was anything doubtful regarding a response, they discussed it together and reached a consensus.

### Outcomes

The primary outcomes were the prevalence of DNR, full life support, and “undecided” decisions among older Thai adults during the COVID-19 pandemic. The secondary outcomes were the reasons for, and the factors associated with these decisions.

### Statistical analysis

Statistical analyses were performed using the software STATA (version 15.1 serial number: 301506318005). We used the *c*hi-squared test and Fisher's exact test for categorical data. For continuous data, we applied one-way analysis of variance for three independent variables. We presented the results for categorical data and as means (±standard deviation) for continuous data.

## Results

**FIG. 1.**
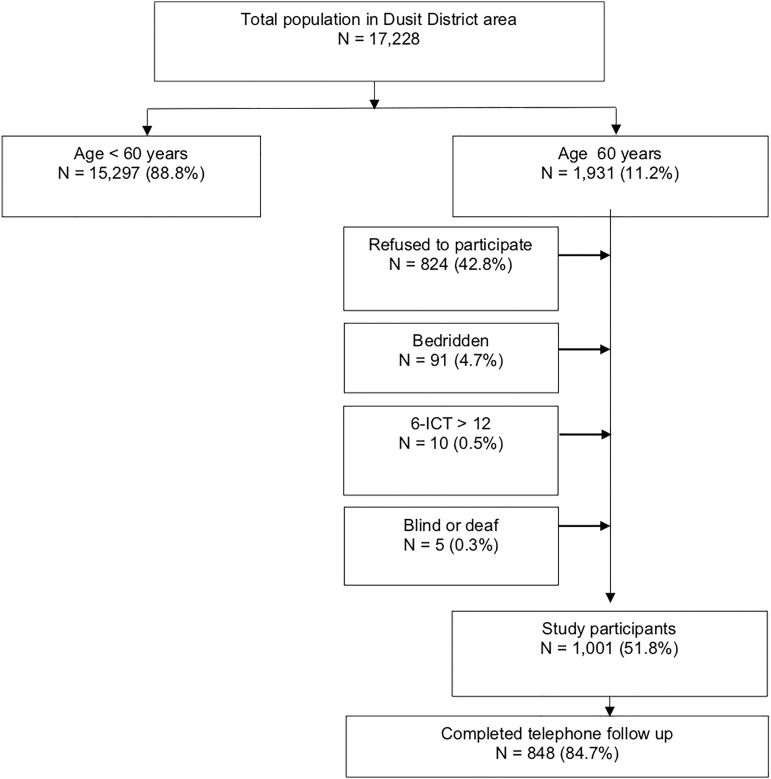
Recruitment and enrollment of participants. 6-CIT, the 6-item cognitive impairment test.

In our primary study, we had 1931 people aged ≥60 years who lived in the Dusit district during our study period. Eighty-hundred twenty-two refused to participate (42.8%), 91 (4.7%) were bedridden, 10 (0.5%) had 6-CIT scores greater than 12, and 5 were blind or deaf. At the end, 1001 older adults participated in the third month's survey, and 848 completed the data via the telephone follow-up (Fig. 1).

Demographic data are presented in Table 1. The prevalence of DNR decisions was 49.8% (422/848) and that of full life support decisions was 44.5% (377/848). However, 5.8% (49/848) were undecided. The mean age of the participants was 70.5 (±6.7) years.

**Table 1.** Baseline Characteristics

Characteristic	Total, *n* = 848 (%)	DNR, *n* = 422 (%)	Full life support, *n* = 377 (%)	Undecided, *n* = 49 (%)	*p*
Gender					
Female	585 (69.0)	293 (69.4)	255 (67.6)	37 (75.5)	0.513
Age (years), mean (SD)	70 (56.7)	70.9 (6.8)	70.1 (6.7)	69.5 (6.3)	0.689
60–74	626 (80.2)	302 (71.6)	285 (75.6)	39 (79.6)	0.681
75–84	191 (17.2)	103 (24.4)	79 (20.9)	9 (18.4)	
≥85	31 (2.6)	17 (4.0)	13 (3.5)	1 (2.0)	
Religious					
Buddhism	835 (98.5)	418 (99.1)	370 (98.1)	47 (95.9)	0.100
Christianity	2 (0.2)	1 (0.2)	1 (0.2)	0	
Islam	10 (1.2)	3 (0.7)	6 (1.6)	1 (2.0)	
Sikhism	1 (0.1)	0	0	0	
Education level					
Less than high school	539 (63.6)	277 (65.6)	227 (60.2)	35 (71.4)	0.419
High school	264 (31.1)	125 (29.6)	127 (33.7)	12 (24.5)	
College or higher	45 (5.3)	20 (4.7)	23 (6.10)	2 (4.1)	
6-CIT score, mean (SD)	5.91 (3.3)	6.06 (3.4)	5.72 (3.1)	6.14 (3.3)	0.134
Disability	55 (6.5)	31 (7.4)	22 (5.8)	2 (4.1)	0.644
Monthly income (Thai Baht), mean (SD)	6029 (9262)	6367 (11557)	5657 (6277)	5944 (5619)	0.844
Professional					0.708
Unemployee	433 (51.1)	215 (51.0)	192 (50.9)	26 (53.1)	
General trading career	153 (18.0)	80 (19.0)	62 (16.5)	11 (22.5)	
Employment	132 (15.6)	67 (15.9)	57 (15.1)	8 (16.3)	
Others	87 (10.3)	37 (8.8)	47 (12.5)	3 (6.1)	
Retired government employee	43 (5.1)	23 (5.5)	19 (5.0)	1 (2.0)	
Has care giver	659 (77.7)	329 (78.0)	290 (76.9)	40 (81.6)	0.746
Age of care giver (years), mean (SD)	52.9 (17.2)	53.7 (17.6)	52.1 (17.0)	51.2 (15.2)	0.649
Used walking aid	77 (9.1)	43 (10.2)	32 (8.5)	2 (4.1)	0.321
CCI score, mean (SD)	3.1 (1.1)	3.1 (1.1)	3.0 (1.1)	2.9 (0.9)	0.121
Hypertension	480 (56.6)	227 (53.8)	230 (61.0)	23 (46.9)	0.045
Diabetes	201 (23.7)	93 (22.0)	96 (25.5)	12 (24.5)	0.519
Dementia	111 (13.1)	63 (14.9)	40 (10.6)	8 (16.3)	0.866
Myocardial infarction	33 (3.9)	15 (3.6)	17 (4.5)	1 (2.0)	0.045
Cerebrovascular accident or transient ischemic attack	12 (1.4)	9 (2.1)	3 (0.8)	0	0.445
Congestive heart failure	5 (0.6)	3 (0.7)	2 (0.5)	0	1.000
Chronic obstructive pulmonary disease	3 (0.4)	1 (0.2)	1 (0.3)	1 (2.0)	0.164
Localized solid tumor	3 (0.4)	1 (0.2)	2 (0.5)	0	0.669
Mild liver disease	2 (0.2)	1 (0.2)	1 (0.3)	0	1.000
Moderate to severe chronic kidney disease	2 (0.2)	2 (0.5)	0	0	0.557
Lymphoma	2 (0.2)	1 (0.2)	1 (0.3)	0	1.000
Metastasis solid tumor	2 (0.2)	1 (0.2)	1 (0.3)	0	1.000
Alcohol	70 (8.3)	35 (8.3)	34 (9.0)	1 (2.0)	0.257
Smoking	67 (7.9)	33 (7.8)	30 (8.0)	4 (8.2)	1.000
Polypharmacy (≥5)	173 (20.4)	79 (18.7)	89 (23.6)	5 (10.2)	0.042
Frailty (score ≥3)	254 (30.0)	124 (29.4)	114 (30.2)	16 (33.7)	0.885
Barthel index ADL					
Independent	844 (99.5)	420 (99.5)	375 (99.5)	49 (100)	1.000
WHOQOL-BREF-THAI, mean (SD)	98 (10)	98 (12)	98 (11)	98 (11)	0.686
Score 96–130: Good QOL	506 (59.7)	248 (58.8)	227 (60.2)	31 (63.3)	0.768
Score 61–95: Medium QOL	341 (40.2)	174 (41.2)	149 (39.5)	18 (36.7)	
Score 26–60: Poor QOL	1 (0.12)	0	1 (0.27)	0	

6-CIT, the 6-item cognitive impairment test; ADL, activity of daily living; CCI, Charlson comorbidity index; DNR, do-not-attempt resuscitation; SD, standard deviation; WHOQOL-BREF-THAI, World Health Organization Quality of Life.

We separated the participants into three groups: (1) do-not-resuscitate, (2) full life support, and (3) undecided groups.

### Knowledge and perception of COVID-19 among participants

The percentage of participants who reported that they knew about COVID-19 was 99.2%, and 58.7%, 57.43%, and 51.8% understood that the routes of transmission of COVID-19 were contact, airborne, and droplets, respectively (Table 2). One-fifth (19.1%) had a history of traveling outside Bangkok in the past 14 days, and participants in the “full life support” and “undecided” groups reported a higher frequency of travels in the past 14 days than those in the DNR group. Only three participants (0.4%) knew that there were confirmed cases of COVID-19 in their communities.

**Table 2.** Awareness and Perception of COVID-19 in Older Adults

Perception about COVID-19	Total, *N* = 848 (%)	DNR, *N* = 422 (%)	Full life support, *N* = 377 (%)	Undecided, *N* = 49 (%)	*p*-
Have you ever heard of and known about COVID-19?	841 (99.2)	418 (99.21)	374 (99.2)	49 (100)	1.000
Route of transmission					
Contact	498 (58.7)	252 (59.7)	221 (58.6)	25 (51.0)	0.503
Airborne	487 (57.4)	235 (55.3)	220 (58.4)	32 (65.3)	0.396
Droplet	439 (51.8)	223 (52.8)	196 (52.0)	20 (40.8)	0.278
History of traveling in past 14 days	162 (19.1)	63 (14.8)	85 (22.6)	14 (28.6)	0.005
Confirm COVID-19 cases in your community	3 (0.4)	2 (0.5)	1 (0.3)	0	0.578

### Reasons for requesting a “do-not-resuscitate” order

The three most common reasons participants reported requesting a DNR order in case of infection of COVID-19 and development of cardiac arrest were (Table 3): “I'm too old” in 232 (55.0%) participants; followed by, “sometimes you need to let things go and let me die naturally” in 66 (15.6%) participants; and finally, “I don't want any pain,” in 36 (8.5%) participants.

**Table 3.** Reason for Patients' Decision to Issue a “Do-Not-Attempt-Resuscitation” Order If Infected with COVID-19 and Have Suffered Cardiac Arrest

Reason	*n* (%)
I'm too old	232 (55.0)
Sometimes you need to let things go and let me die naturally	66 (15.6)
I don't want any pain	36 (8.5)
I have no reason	25 (5.9)
I feel that I will be a burden on family and others	23 (5.5)
I feel that I might infect others	12 (2.8)
I feel very lonely because I live on my own	9 (2.1)
I feel that I'm not going back to normal	8 (1.9)
I h*ave* ma*de* a decision with my relatives not *to* resuscita*te*	6 (1.4)
I have multiple diseases	4 (1.0)
I don't have *any* money	1 (0.2)

### Reasons for requesting “full life support” in case of cardiac arrest after contracting COVID-19 infection

The three most common reasons reported by the “full life support” group for requesting full life support were (Table 4): “I don't want to die” in 195 (51.7%) participants; followed by, “If there is any chance for me to survive, please don't let me die” in 97 (25.7%) participants; and “I have no reasons” in 34 (9.0%) participants.

**Table 4.** Reason for Patients' Decision to Request “Full Life Support” If Infected with COVID-19 and Have Suffered Cardiac Arrest

Reason	*n* (%)
I don't want to die	195 (51.7)
If there is any chance to be survive, please don't let me die	97 (25.7)
I have no reason	34 (9.0)
I have a family to take care of	20 (5.3)
I have made a decision with my relatives to resuscitate	16 (4.2)
When the time comes, the doctor has the responsibility as to whether to resuscitate or not	14 (3.7)
Now I'm not infected	1 (0.3)

### Reasons given by the “undecided” patients for chosing no option in case they developed cardiac arrest after contracting COVID-19 infection

The three most common reasons given by the “undecided” group for their choice were (Table 5): “Now, I'm not infected” in 13 (26.5%) participants, “I have made a decision with my relatives to make a decision for me (to resuscitate or not resuscitate)” in 12 (24.5%) participants, and “I have no reasons” in 12 (24.5%) participants.

**Table 5.** Reason for Patients Who Are “Undecided” If Infected with COVID-19 and Have Suffered Cardiac Arrest

Reason	*n* (%)
Now I'm not infected	13 (26.5)
I have made a decision with my relatives to make a decision for me (to resuscitation or not resuscitation)	12 (24.5)
I have no reason	12 (24.5)
When the time comes, the doctor has the responsibility to make a decision whether to resuscitate or not	9 (18.4)
I have multiple diseases	3 (6.1)

In our study, none of the factors evaluated (frailty phenotype, educational level, severe illness, or cancer) was associated with the DNR decision (Table 1).

## Discussion

In this study, nearly half of the surveyed older Thai adults completed a DNR order for the situation of infection with COVID-19, during the early stages of the pandemic. This contrasts the findings of Jihae Lee's study on end-of-life decisions of patients during the COVID-19 era,^13^ who reported a prevalence of 17.3% of DNR decisions among critically ill New York patients. However, most of the participants in that study were already either infected or at high risk of COVID-19 infection.^14^ After the subjects had encountered palliative care, the prevalence of DNR decisions increased dramatically to more than 90%.^14^

In this study, the prevalence of DNR orders was higher than that in other studies among healthy older adults. Chan CWH's study showed that 18.3% of Hong Kong patients, among whom the majority were healthy older adults, had given advance directives.^30^ Further, one study in Japan found that 12% of older Japanese adults had a palliative goals-of-care plan.^31^ The results of this study are similar to those of Srinonprasert et al. They carried out their study in the outpatient clinics of two hospitals offering palliative care, situated in northeast Bangkok (at the center of Thailand).

They found that 59.2% of older Thai adults wished to die at home.^18^ However, the context of our study substantially differs from theirs as the chances of survival were minimal due to their critical illnesses or backgrounds of chronic illness.

Spirituality and religion may influence these decisions. Almost the entire Thai population are Buddhists. Thai Buddhists perceive death as a natural phenomenon in life; to them, death cannot be controlled and can occur at any time. Letting everything go before dying is central to finding calmness. Further, Buddhists practice “clean living,” and acquiring merit and additional means for attaining a peaceful death and a peaceful life before death is highly valued.^32^

During the early stages of the pandemic, there was much uncertainty about the outcome of the disease and the aggressive measures needed to maintain life in patients infected with COVID-19. The limited information from various sources may have led people to believe that more intensive care was needed in conditions that were very likely to lead to death. This did not align with their religious beliefs and may have prompted many to request a DNR order. Understanding the natural history of COVID-19 and its treatment is, therefore, important.

In our study, even though most of the participants claimed that they knew about COVID-19, only half could correctly state the exact routes of transmission.^33^ Also, participants in the “undecided” group traveled more frequently (in the past 14 days) during the early months of the pandemic. According to the World Health Organization, routes of transmission such as droplet transmission involve close contact between people. Traveling may give participants more risk to come into contact with people. This indicates that our population may have had insufficient knowledge of COVID-19. COVID-19 impacts mental health and can cause significant worry and fear.^34^

A better understanding of the disease may modify end-of-life decisions. Knowledge of the mortality rate, treatment opportunities, routes of transmission, and factors that may increase the risk of deterioration in infected patients could change end-of-life decisions.

Steinhauster KE's study^35^ showed that freedom from pain was more of a priority than “being at peace with God” and “the presence of family.” Their study found significant spiritual and religious factors interplaying with medical decision making.^35,36^ There are several levels of mindfulness and beliefs in Buddhism. However, even considering the fact that the majority of participants in the “full life support” group were Buddhist, the most common reason they quoted was “I don't want to die.” Therefore, even though they are Buddhist, they probably experience fear in their mind.

Interestingly, in a crude analysis, our study found that hypertension and a history of myocardial infarction were associated with full life support decisions. There is lack of evidence to support that these factors are likely to be found in the full life support group. Owing to the low population of participants with each factor and the fact that the *p*-values were close to 0.05, this could be the result of chance. However, hypertension is a common chronic disease; therefore, older adults may be familiar with it and may not perceive it as a comorbidity.

The low prevalence of a history of myocardial infarction in the palliative care group concords with the results of a previous study.^37^ Another reason may be that patients could perceive these factors as wellness control and health consciousness; therefore, they could be acquainted with related medical operative procedures and be hopeful of surviving. Other physical performance factors (including 6-CIT, disability, use of walking aids, Barthel index, ADL score, and frailty score) were not associated with the DNR decision because of the low number of participants with poor functional status in our study.

Future studies should include an in-depth examination of participant lifestyles, such as family life expectancy, and religious affiliations and beliefs, to better understand their end-of-life decisions.

### Limitations

There are several limitations in our study. First, due to the cross-sectional nature of the study, the decisions of the participants could be temporary and could change over time. Second, because we used a telephone survey, cases of partial hearing loss and electric technical problems were common; these may have interfered with the answers the patients gave. Third, the question “Have you heard about COVID-19?” is nonspecific; participants may have heard about COVID-19 without exactly understanding the disease.

Our study included only a few assessment questions to confirm this knowledge. Fourth, the limitations of the health system, particularly in Bangkok, could have contributed to the results of this study. The prevalence could be misleadingly high because of the pandemic and the methodological limitations of the process of coding the reasons for the decisions. Fifth, this was a single-center study; therefore, the results may not be generalizable. Given that the choice of the DNR order is not a simple question to ask, RAs with less experience in the field may have altered the results.

In addition, we used the 6-CIT to test cognitive functions; this test has not been validated in older Thai adults. Participants with mild cognitive impairment may not have truly understood the questions. Further, owing to the small number of variables, no regression analysis was performed in this study to determine the association between factors. Finally, spirituality and religion, which were not evaluated in our study, may be important factors associated with end-of-life decisions in older Thai adults.

## Conclusions

In conclusion, our study found that almost half of the older Thai adults requested a do-not-resuscitate order in case they develop cardiac arrest after contracting COVID-19 infection. The most common reasons given for requesting a DNR order were old age, acceptance of death, and fear of pain. Future studies should consider spirituality and religion as significant factors that could influence end-of-life decisions.

## Data Availability

All data generated and/or analyzed during the current study were available from the corresponding author on reasonable request.

## Abbreviations Used

6-CIT: the 6-item cognitive impairment test
ADL: activity of daily living
CCI: Charlson comorbidity indices
COVID-19: coronavirus disease 2019
CPR: cardiopulmonary resuscitation
DNR: do-not-attempt resuscitation
ED: emergency department
EPs: emergency physicians
RA: research assistant
SD: standard deviation
WHOQOL-BREF-THAI: World Health Organization Quality of Life
